# Prevalence and Associated Factors of Lupus in the United States: Third National Health and Nutritional Examination Survey (NHANES III)

**DOI:** 10.3389/fmed.2020.00213

**Published:** 2020-05-27

**Authors:** Xiaomei Leng, Jin Xia, Xiaofeng Zeng, Yiqing Song

**Affiliations:** ^1^Key Laboratory of Rheumatology and Clinical Immunology, Ministry of Education, Department of Rheumatology and Clinical Immunology, Peking Union Medical College Hospital, Chinese Academy of Medical Sciences & Peking Union Medical College, National Clinical Research Center for Dermatologic and Immunologic Diseases (NCRC-DID), Beijing, China; ^2^Department of Epidemiology, Richard M. Fairbanks School of Public Health, Indiana University, Indianapolis, IN, United States

**Keywords:** lupus, NHANES, risk factor, protective factor, lutein, calcium

## Abstract

This study aimed to examine the prevalence and associated factors of lupus among adults in the United States. This study included 20,045 participants aged 17 years and older from the Third National Health and Nutritional Examination Survey (NHANES III) from 1988 to 1994. Their lupus status was determined by survey questions in terms of a clinician's diagnosis. Demographics and laboratory test results of all participants were collected, including biochemistry, nutrition, and antibody biomarkers. Continuous variables were compared between cases with reported lupus and non-case controls by *t*-test, while the Chi-square test was used for categorical variables. Weighted multivariate-adjusted logistic regression models after adjustment of covariates were used to identify associated factors of lupus risk. Of 20,045 participants, 40 people who self-reported a lupus diagnosis were identified, giving a prevalence of 241 per 100,000 (*n* = 40; 95% confidence interval: 133–349 per 100,000). Many factors differed significantly between lupus cases and controls. Multivariate logistic regression analysis further identified previous and current smoking along with elevated serum levels of chloride, globulin, lactate dehydrogenase, uric acid, cholesterol, and lutein or zeaxanthin as risk factors; while protective factors against lupus included non-white race, obesity, elevated serum levels of bicarbonate, creatinine, total calcium, and vitamin B12, as well as elevated urinary albumin and iodine. Our nationwide data indicate that race, obesity, cigarette smoking, and certain biomarkers such as serum lutein or zeaxanthin, calcium, and cholesterol may be associated with the development or progression of lupus, although these findings need to be confirmed in further prospective investigations.

## Introduction

Lupus is a chronic autoimmune disease mainly affecting women (1). The prevalence of lupus ranges from 6.5 to 178.0 per 100 000 worldwide. Prevalence is higher in adult non-Caucasian women, possibly due to genetic, environmental, and demographic factors (2). Previous studies demonstrated that age is a key factor associated with the manifestations and prognosis in cases of lupus. Though lupus can develop at any age, the mean age of diagnosis among adults varies from 24 to 32 years (3, 4). Again, females are more likely to develop lupus, especially during theirchild-bearing years (2). One previous study found that Amerindian ancestry may be positively related to the presence of more risk alleles forlupus (5). Previous studies also demonstrated that tuberculosis, serum death decoy receptor 3, and smoking are also risk factors (6–8). There is also strong evidence that increased risk of lupus may be associated with exposure to crystalline silica, use of oral contraceptives, and postmenopausal hormone replacement therapy (9). Additionally, exposure to solvents, residential and agricultural pesticides, air pollution, and heavy metals may be also associated with risk (9). Though one study indicated that vitamin D deficiency may be an important risk factor (10),which serum measures increase risk or offer protection remains unclear, especially serum nutritional factors.

This study aimed to comprehensively examine the national prevalence of lupus as well as its risk factors in adults of the United States based on the representative population from the Third National Health and Nutrition Examination Survey (NHANES III).

## Materials and Methods

### Study Participants

We used NHANES III (1988–1994) data to explore the risk factors for lupus. NHANES III is a nationally representative sample of the civilian non-institutionalized population aged 2 months and older in the United States. Health information and laboratory data on the population were collected through household interviews and medical examinations based on a multi-stage, stratified, and clustered sample design. Our study is restricted to adults only. A total of 20,050 participants aged 17 years and older completed both the household interviews and the medical examinations. Of them, five people with unknown lupus status were excluded from the study. As a result, a total sample of 20,045 participants was available for our analysis.

This study was only involved in the secondary data analysis of existing U.S. national database that are publicly available and have been de-identified. This research qualified for exemption of IRB Human Subjects approval under 45 CFR 46.101(b) (4) as specified by the Federal Regulations for Protection of Human Research Subjects. Thus, this is an exempt study and there is no need for an IRB approval from our institution.

### Outcome of Interest

Lupus status of the examinees was ascertained through the survey questions, “Has a doctor ever told you that you had lupus?”

### Risk Factors

In NHANES III, blood and urine specimens were collected at mobile examination centers and analyzed using standardized laboratory test procedures, including biochemistry, nutritional, and antibody biomarkers. Laboratory test results in SI units were used. Self-reported health conditions, including cardiovascular disease (CVD), cancer, arthritis, thyroid disease, and metabolic syndrome, were collected through questionnaires. Metabolic syndrome was defined as the presence of three or more of the five following features: (1) blood pressure ≥130 mmHg systolic blood pressure (SBP) or ≥85 mmHg diastolic blood pressure (DBP) or antihypertensive drug therapy; (2) fasting glucose ≥100 mg/dL or drug treatment for hyperglycemia; (3) high-density lipoprotein cholesterol (HDL-C) <40 mg/dL in men and <50 mg/dL in women; (4) fasting triglycerides ≥150 mg/dL; and (5) waist circumference >102 cm in men and >88 cm in women (11, 12).

### Covariates

Age, sex, race (White or Non-white), urbanization, education, smoking status (never, former, or current), and physical activity were self-reported. Specifically, physical activity was assessed using metabolic equivalent (MET) intensity levels. Weight and height were measured during the physical examination. Body mass index (BMI) was computed as weight (kg) divided by height (m^2^) and categorized into four groups: underweight (BMI <18.5 kg/m^2^), normal (BMI of 18.5–24.9 kg/m^2^), overweight (BMI of 25–29.9 kg/m^2^), or obese (BMI ≥ 30 kg/m^2^). Serum biochemical and nutritional biomarkers as well as urine biomarkers and antibodies were also measured.

### Statistical Analysis

We compared demographic and biochemical characteristics and health conditions between participants with and without lupus using the *t*-test for continuous variables and the Chi-square test for categorical variables, accounting for complex sample survey designs. The association between risk factors and lupus was expressed as an odds ratio (OR) with a 95% confidence interval (CI) from the logistic regression models. In the univariate analysis, a crude model was first constructed using only age, sex, race, BMI, and smoking status. All biomarkers and health conditions were individually investigated based upon the crude model, except for serum FSH (follicle-stimulating hormone) and serum luteinizing hormone, which were assessed among women only. From these results, the variables that met the criterion of *p* < 0.1 were selected for stepwise regression analysis to identify the most important risk factors for lupus. SAS macros for stepwise selection were developed to analyze data from a complex multi-staged probability survey (13). The selection criterion to add or remove variables was set at 0.15 in a stepwise procedure. Similar to the univariate analysis, the same covariates were adjusted in the multivariate regression analysis. For the final multivariate model, the receiver operating characteristic (ROC) curve was used to assess the model accuracy (Supplemental Figure 1). Appropriate sample weights as provided in the NHANES data files were used for all analyses. A two-tailed statistical significance level of 0.05 was established. All analyses were performed in SAS version 9.4 (SAS Institute, Cary, North Carolina, USA).

## Results

A total of 20,045 participants were finally included in our analysis. The prevalence of lupus was 241 per 100,000 (*n* = 40; 95% confidence interval [CI]: 133–349 per 100,000), which was very similar to the previous report (14). After accounting for the survey design, the mean age was significantly older in the lupus group than in the non-lupus group (50.26 vs. 43.23 years, *P* = 0.04). The percentage of people living in urban areas in the lupus group was significantly higher than that in the non-lupus group (73.03 vs. 49.67%, *P* = 0.01). The percentage of never smoking people in the lupus group was significantly lower than that non-lupus group (27.22 vs. 47.25%, *P* = 0.03). Total mean MET frequency in the non-lupus group was significantly higher than that in the lupus group (24.37 vs. 10.05, *P* < 0.01). Prevalence of arthritis and CVD were all significantly higher in the lupus group than that in the non-lupus group (28.00 vs. 7.51%, *P* < 0.01; 20.10 vs. 5.66%, *P* = 0.02). No significant difference between the two groups were identified in terms of other characteristics (Table 1).

**Table 1:** Demographic and characteristics of NHANES III participants by lupus status (*N* = 20,045).

**Characteristics**	**Lupus group (*N* = 40)**	**No lupus group (*N* = 20,005)**	***P*-value**
**Demographic**			
Gender, Female (*N*, %)	32 (74.77%)	10,616 (52.18%)	0.10
Age, years (mean, SD)	50.26 (0.93)	43.23 (0.40)	0.04*
Urban area (*N*, %)	28 (73.03%)	9,949 (49.67%)	0.01*
**Census region (*****N*****, %)**			
Northeast	6 (20.65%)	2,924 (20.75%)	0.47
Midwest	8 (16.62%)	3,845 (24.07%)	
South	14 (28.64%)	8,542 (34.28%)	
West	12 (34.08%)	4,694 (20.90%)	
Education, years (mean, SD)	12.94 (0.08)	12.24 (0.08)	0.18
**Race (*****N*****, %)**			
White	27 (89.76%)	13,707 (84.26%)	0.20
Nonwhite	13 (10.24%)	6,298 (15.74%)	
**Smoking (*****N*****, %)**			
Never	16 (27.22%)	10,217 (47.25%)	0.03*
Previous	12 (50.96%)	4,794 (24.49%)	
Current	12 (21.82%)	4,976 (28.26%)	
**BMI (*****N*****, %)**
Underweight	2 (1.00%)	460 (2.78%)	0.86
Normal	14 (47.51%)	7,061 (43.83%)	
Overweight	13 (35.32%)	6,101 (31.69%)	
Obese	8 (16.16%)	4,435 (21.71%)	
Total metabolic equivalents (MET) frequency (weighted mean, weighted SD)	10.05 (0.67)	24.37 (4.64)	<0.01**
**Health conditions**			
CVD (*N*, %)	6 (20.10%)	1,734 (5.66%)	0.02*
Cancer (*N*, %)	6 (16.59%)	1,497 (7.18%)	0.09
Arthritis (*N*, %)	15 (28.00%)	1,650 (7.51%)	<0.01**
Thyroid disease (*N*, %)	5 (9.97%)	932 (4.79%)	0.20
**Metabolic syndrome (*****N*****, %)**			
Yes	10 (17.18%)	4,943 (21.02%)	0.64
No	30 (82.82%)	15,062 (78.98%)	

*Weighted statistics (including percentage, mean, and SD) were reported. T-test and Chi-square test were conducted by accounting for complex sampling design*.

NHANES, National Health and Nutritional Examination Survey; SD, standard deviation; BMI, body mass index; MET, metabolic equivalent; CVD, cardiovascular disease. ^*^P < 0.05; ^**^P < 0.01.

Serum levels of alanine aminotransferase, bicarbonate, glucose, total calcium, and urinary iodine in lupus group were all significantly lower than in the non-lupus group. Serum levels of lactate dehydrogenase, globulin, and cholesterol were all significantly higher in the lupus group (Table 2).

**Table 2:** Biochemistry profile of NHANES III participants by lupus status (*N* = 20,045).

**Characteristics**	**Lupus group (*N* = 40)**	**No lupus group (N = 20,005)**	***P*-value**
**Biochemistry (mean, SD)**			
Serum alanine aminotransferase: SI (U/L)	13.30 (0.88)	17.60 (0.40)	<0.01**
Serum gamma glutamyl transferase: SI(U/L)	26.51 (1.38)	29.08 (0.57)	0.51
Serum aspartate aminotransferase: SI (U/L)	21.84 (0.34)	21.40 (0.19)	0.70
Serum albumin: SI (g/L)	39.81 (0.16)	41.93 (0.21)	0.07
Serum bicarbonate: SI (mmol/L)	26.62 (0.43)	28.13 (0.23)	0.02*
Serum alkaline phosphatase: SI (U/L)	78.61 (2.04)	83.15 (0.75)	0.31
Serum lactate dehydrogenase: SI (U/L)	174.46 (3.79)	157.11 (2.03)	0.04*
Serum total protein: SI (g/L)	74.65 (0.58)	73.10 (0.16)	0.07
Serum globulin: SI (g/L)	33.83 (0.37)	31.56 (0.23)	<0.05*
Serum creatinine: SI (umol/L)	97.99 (4.83)	94.64 (0.29)	0.59
Serum cholesterol: SI (mmol/L)	5.93 (0.09)	5.34 (0.02)	0.03*
Serum glucose: SI (mmol/L)	5.07 (0.07)	5.36 (0.04)	0.04*
Serum total bilirubin: SI (umol/L)	9.50 (0.23)	10.56 (0.11)	0.31
Serum total calcium: SI (mmol/L)	2.28 (0.005)	2.32 (0.01)	0.04*
Serum C-reactive protein (mg/dL)	0.51 (0.03)	0.41 (0.01)	0.40
Serum FSH: SI (IU/L)	30.72 (4.47)	29.05 (0.89)	0.56
Serum luteinizing hormone: SI (IU/L)	10.32 (1.58)	12.21 (0.35)	0.61
Lead: SI (umol/L)	0.18 (0.01)	0.17 (0.005)	0.57
Serum transferrin saturation (%)	22.85 (0.38)	26.31 (0.21)	0.07
Urinary albumin (ug/mL)	233.88 (17.53)	26.52 (1.40)	0.22
Urinary cadmium: SI (nmol/L)	8.00 (0.78)	5.71 (0.14)	0.15
Urinary creatinine: SI (mmol/L)	10.17 (1.09)	11.63 (0.11)	0.53
Urinary iodine (ug/dL)	12.61 (0.70)	23.63 (2.13)	<0.01**

*Weighted statistics (including percentage, mean, and SD) were reported. T-test was conducted by accounting for complex sampling design*.

NHANES, National Health and Nutritional Examination Survey; SD, standard deviation. ^*^P < 0.05; ^**^P < 0.01.

Serum latex antibody was significantly lower in the lupus group while serum cholesterol was also significantly higher in the lupus group (Table 3).

**Table 3:** Urine and antibody test of NHANES III participants by lupus status (*N* = 20,045).

**Characteristics**	**Lupus group (*N* = 40)**	**No lupus group (*N* = 20,005)**	***P*-value**
**Nutrition Biomarkers (mean, SD)**			
Plasma fibrinogen: SI (g/L)	3.21 (0.06)	3.05 (0.03)	0.28
Serum beta carotene: SI (umol/L)	0.37 (0.03)	0.37 (0.01)	0.99
Serum beta cryptoxanthin: SI (umol/L)	0.19 (0.01)	0.16 (0.00)	0.38
Serum cholesterol: SI (mmol/L)	5.87 (0.09)	5.23 (0.02)	0.01*
Serum lutein/zeaxanthin: SI (umol/L)	0.49 (0.02)	0.38 (0.01)	0.17
Serum selenium: SI (nmol/L)	1.60 (0.02)	1.59 (0.01)	0.81
Serum vitamin A: SI (umol/L)	1.97 (0.04)	2.04 (0.01)	0.40
Serum vitamin B12: SI (pmol/L)	321.15 (1.14)	356.74 (1.18)	0.45
Serum vitamin C: SI (mmol/L)	39.96 (1.87)	42.54 (0.87)	0.67
Serum vitamin E: SI (umol/L)	28.24 (0.81)	26.54 (0.23)	0.24
**Antibody tests**			
Serum rubella antibody (IU) (mean, SD)	121.14 (7.55)	104.78 (1.81)	0.59
Serum tetanus antibody (U/mL) (mean, SD)	0.77 (0.29)	1.02 (0.03)	0.43
Serum varicella antibody (mean, SD)	14.69 (0.98)	12.78 (0.19)	0.18
Serum toxoplasmosis antibody (mean, SD)	40.19 (17.89)	22.37 (1.10)	0.42
Serum latex antibody (IU/mL) (mean, SD)	0.20 (0.01)	0.56 (0.01)	<0.01**
**Serum hepatitis A antibody (N, %)**			
Negative	19 (47.63%)	7435 (64.50%)	0.16
Positive	18 (52.37%)	9126 (35.50%)	
**Serum hepatitis B core antibody (N, %)**			
Negative	34 (81.12%)	15259 (94.00%)	0.09
Positive	2 (18.88%)	1310 (6.00%)	

*Weighted statistics (including percentage, mean, and SD) were reported. T-test was conducted by accounting for complex sampling design*.

NHANES, National Health and Nutritional Examination Survey; SD, standard deviation. ^*^P < 0.05; ^**^P < 0.01.

Results from the crude model indicated that age was the only significant risk factor for lupus (OR = 1.03, 95% CI: 1.01–1.04) (Table 4). However, multivariate logistic regression analysis demonstrated that, after adjusted age, sex, race, BMI and smoking status, non-white race (OR = 0.35, 95% CI: 0.21–0.59), and obese (OR = 0.33, 95% CI: 0.26–0.40) were all significantly protective factors of people with reported lupus, while previous smoking (OR = 6.75, 95% CI:2.83–16.11)and current smoking (OR = 3.87, 95% CI:1.20–12.45) were risk factors for lupus (Table 4).

**Table 4:** Logistic regression models of demographics for lupus from NHANES III (*N* = 20,045).

**Variable**	**Univariate risk factor**		**Multivariate risk factors**	
	**Odds ratio**	**95% CI**	**Odds ratio**	**95% CI**
**Demographic characteristics**				
Age (yr)	1.03**	(1.01, 1.04)	1.01	(0.99, 1.03)
Nonwhite race	0.66	(0.28, 1.55)	0.35**	(0.21, 0.59)
Female sex	2.41	(0.66, 8.78)	0.95	(0.60, 1.52)
**Smoking**				
Never	1	–	1	–
Previous	2.91	(0.95, 8.86)	6.75**	(2.83, 16.11)
Current	1.29	(0.33, 5.13)	3.87*	(1.20, 12.45)
**BMI**				
Underweight	0.32	(0.06, 1.80)	0.26	(0.03, 1.91)
Normal	1	–	1	–
Overweight	1.002	(0.33, 3.09)	0.61	(0.34, 1.08)
Obese	0.65	(0.16, 2.59)	0.33**	(0.26, 0.40)
Presence of metabolic syndrome	0.52	(0.14, 1.90)	–	–

NHANES, National Health and Nutritional Examination Survey; CI, confidence interval; BMI, body mass index. ^*^P < 0.05; ^**^P < 0.01.

In the univariate logistic regression analysis of biochemistry profiles, only serum bicarbonate (OR = 0.88, 95% CI: 0.79–0.97) was found to be protective against lupus, while serum chloride (OR = 1.11, 95% CI:1.01–1.22), serum creatinine (OR = 1.003, 95% CI:1.00–1.01), serum globulin (OR = 1.11, 95% CI: 1.07–1.16), serum lactate dehydrogenase (O = 1.006, 95% CI: 1.003–1.01), and serum uric acid (OR = 1.005, 95% CI: 1.00–1.01) were all revealed to be risk factors.

Based on the results from the univariate logistic regression analysis, we included all variables which met the criterion for inclusion as primary variables for the stepwise regression analysis. Of the possible biochemical, urinary, and antibody biomarkers, twelve variables with *p* ≤ 0.05 were identified as the associated factors of lupus with adjustment of its conventional risk factors (Tables 4–6). Results from the final multivariate logistic regression model demonstrated that, after adjusting for age, sex, race, BMI, and smoking status, non-white race (OR = 0.35, 95% CI: 0.21–0.59), and obesity (OR = 0.33, 95% CI: 0.26–0.40) were both protective factors of people with reported lupus, while previous smoking (OR = 6.75, 95% CI: 2.83–16.11) and current smoking (OR = 3.87, 95% CI: 1.20–12.45) were risk factors for lupus (Table 4). Serum bicarbonate (OR = 0.96, 95% CI: 0.95–0.98), serum creatinine (OR = 0.97, 95% CI: 0.96–0.99), and serum total calcium (OR = 0.18, 95% CI: 0.04–0.77) were all protective against lupus, while serum chloride (OR = .14, 95% CI: 1.08–1.19), serum globulin (OR = 1.18, 95% CI: 1.13–1.23), serum lactate dehydrogenase (OR = 1.009, 95% CI: 1.007–1.01), and serum uric acid (OR = 1.004, 95%: 1.002–1.01) were all risk factors. For the nutritional biomarkers, only serum vitamin B12 (OR = 0.999, 95% CI: 0.998–1.00) was protective against lupus, while both serum cholesterol (OR = 1.67, 95% CI: 1.15–2.44) and serum lutein/zeaxanthin (OR = 3.81, 95% CI: 2.46–5.90) were risk factors (Table 5).

**Table 5:** Logistic regression models of biochemistry profile for lupus from NHANES III (*N* = 20,045).

**Biochemistry profile**	**Univariate risk factor**		**Multivariate risk factors**	
	**Odds ratio**	**95% CI**	**Odds ratio**	**95% CI**
Serum bicarbonate: SI (mmol/L)	0.88*	(0.79, 0.97)	0.96**	(0.95, 0.98)
Serum chloride: SI (mmol/L)	1.11*	(1.01, 1.22)	1.14**	(1.08, 1.19)
Serum creatinine: SI (umol/L)	1.003*	(1.00, 1.01)	0.97**	(0.96, 0.99)
Serum globulin: SI (g/L)	1.11**	(1.07, 1.16)	1.18**	(1.13, 1.23)
Serum lactate dehydrogenase: SI (U/L)	1.006**	(1.003, 1.01)	1.009**	(1.007, 1.01)
Serum total calcium: SI (mmol/L)	0.06	(0.003, 1.003)	0.18*	(0.04, 0.77)
Serum uric acid: SI (umol/L)	1.005*	(1.00, 1.01)	1.004**	(1.002, 1.01)
Serum cholesterol: SI (mmol/L)	1.39	(0.96, 2.02)	1.67**	(1.15, 2.44)
Serum lutein/zeaxanthin: SI (umol/L)	2.40	(0.93, 6.20)	3.81**	(2.46, 5.90)
Serum vitamin B12: SI (pmol/L)	0.999*	(0.998, 1.00)	0.999*	(0.998, 1.00)

NHANES, National Health and Nutritional Examination Survey; CI, confidence interval. ^*^P < 0.05; ^**^P < 0.01.

**Table 6:** Logistic regression models of urine and antibody tests for lupus from NHANES III (*N* = 20,045).

**Variable**	**Univariate risk factor**		**Multivariate risk factors**	
	**Odds ratio**	**95% CI**	**Odds ratio**	**95% CI**
**Urine tests**				
Urinary albumin (ug/mL)	1.001*	(1.00, 1.001)	0.996**	(0.993, 0.999)
Urinary cadmium: SI (nmol/L)	1.01	(0.99, 1.03)	–	–
Urinary creatinine: SI (mmol/L)	1.02	(0.93, 1.10)	–	–
Urinary iodine (ug/dL)	0.98	(0.95, 1.01)	0.96**	(0.95, 0.97)
**Antibody tests**				
Serum rubella antibody (IU)	1.001	(0.995, 1.01)	–	–
Serum tetanus antibody (U/mL)	0.999	(0.81, 1.24)	–	–
Serum varicella antibody	1.07	(0.98, 1.17)	–	–
Serum toxoplasmosis antibody	1.004	(0.996, 1.01)	–	–
Serum latex antibody (IU/mL)	0.74**	(0.60, 0.90)	–	–
**Serum hepatitis A antibody**				
Negative	1	–	–	–
Positive	1.54	(0.44, 5.43)	–	–
**Serum hepatitis B core antibody**				
Negative	1	–	–	–
Positive	4.24	(0.71, 25.50)	–	–

NHANES, National Health and Nutritional Examination Survey; CI, confidence interval. ^*^P < 0.05; ^**^P < 0.01.

Additionally, both the elevated levels of urinary albumin (OR = 0.996, 95% CI: 0.993–0.999) and urinary iodine (OR = 0.96, 95% CI: 0.95–0.97) were protective against lupus (Table 6).

We also conducted a ROC curve analysis to assess the final model accuracy. We found that the AUC is significantly better than 50% (AUC = 0.69; 95% CI: 0.56–0.83) (Supplemental Figure 1).

## Discussion

This study indicated that risk factors for lupus include previous and current smoking, serum chloride, globulin, lactate dehydrogenase, uric acid, cholesterol, and lutein or zeaxanthin, while among protective factors against lupus are non-white race, obesity, the elevated levels of serum bicarbonate, creatinine, total calcium, vitamin B12, urinary albumin, and iodine.

The nutritional status and food intake of patients with lupus was poor (15). The European Prospective Investigation of Cancer Incidence (EPIC)-Norfolk study demonstrated that zeaxanthin intake was 20% lower in patients with inflammatory polyarthritis (16). Though a previous cohort study found that lutein was not associated with risk of lupus (17), our study found that the elevated level of serum lutein or zeaxanthin may be strong risk factors of lupus. Higher serum levels of lutein or zeaxanthin in lupus patients indicates that lupus may cause decreased metabolic turnover of antioxidants, while the level of serum lutein or zeaxanthin in patients with rheumatoid arthritis was lower than non-RA subjects (18). Since vegetables and corn are rich in lutein and zeaxanthin, individuals on vegetarian diets might be more likely to develop lupus, which needs to be validated by further study.

Our study also found that serum calcium is a protective factor against systemic lupus erythematosus (SLE), possibly decreasing risk by as much as 82%. One previous study found that serum calcium plays an important role in the progression of SLE. Lupus patients are at higher risk of hypocalcemicevents (19), which is consistent with our finding that calcium supplementation may help prevent the progression of SLE. A previous study found that calcium signaling mediated by CD95 can help trafficking of T helper 17 to inflamed organs in mice susceptible to lupus (20). Thus, targeting this pathway may be a potential treatment option for SLE. In T cells of patients with SLE, the modulatory receptors are engaged followed by the modification of calcium signal (21), and calcium responses in lymphocytes increase in SLE (22). As noted above, calcium levels play a critical role in the progression of SLE, placing SLE patients at elevated risk of hypocalcemic events. Abnormal homeostasis of vitamin D and calcium may contribute to SLE and the severity of symptoms (19). Vitamin D deficiency is common in patients with SLE, playing an important role in disease progression (23). A recent meta-analysis found serum levels of vitamin D to be significantly lower in cases than controls, suggesting that vitamin D deficiency may be associated with SLE (24). A systematic review indicated that vitamin D supplementation may decrease levels of inflammatory and hemostatic markers in patients with SLE (25), suggesting that vitamin D supplementation may improve the inflammation of SLE patients (26).

Previous evidence shows that the systemic inflammatory load in patients with lupus may disrupt the homeostasis of cholesterol and increase the tendency toward cholesterol accumulation around cells (i.e., macrophages and endothelium) in the artery wall. The association between the inflammatory state and dyslipidemia in patients with lupus is complicated, involving cholesterol transporters, scavenger receptors, lipoproteins, and oxysterols (27). Our study found that the elevated level of serum cholesterol may be a risk factor of SLE.

Consistent with previous studies, we found smoking to be a risk factor for lupus (7, 8). White race and obesity were protective factors against lupus, contrary to some prior findings (1, 28), though this might be due to differences in study populations or small sample size of people with self-reported lupus. Our study also found that the elevated levels of serum bicarbonate, creatinine, serum vitamin B12, urinary albumin and iodine may be protective factors against SLE, while serum chloride, globulin, lactate dehydrogenase, and uric acid may be risk factors for lupus. However, the effects of these factors were small and need to be validated by further study.

Several limitations of our study should be noted. First, the sample size of people with reported lupus was small: only 40 people with reported lupus were included in the final analysis. Second, the design of our study was retrospective and cross-sectional, and thus incapable of indicating causal association. Additionally, the lupus status of all the people was self-reported, which may have false positive or negative cases, thus bias the results in this study. The recall bias might exist when self-report conditions were included in the analysis.

In conclusion, previous and current smoking, the elevated levels of serum chloride, globulin, lactate dehydrogenase, uric acid, cholesterol, and lutein or zeaxanthin may be risk factors for lupus. Nonwhite race, obesity, the elevated levels of serum bicarbonate, creatinine, total calcium, vitamin B12, urinary albumin and iodine may be protective factors of lupus. A large-scale prospective study is warranted to examine the roles of lutein and zeaxanthin in lupus.

## Data Availability Statement

Publicly available datasets were analyzed in this study. This data can be found here: https://www.cdc.gov/nchs/nhanes/index.htm?CDC_AA_refVal=https%3A%2F%2Fwww.cdc.gov%2Fnchs%2Fnhanes.htm.

## Ethics Statement

This study was only involved in the secondary data analysis of existing U.S. national database that are publicly available and have been de-identified. This research qualified for exemption of IRB Human Subjects approval under 45 CFR 46.101(b) (4) as specified by the Federal Regulations for Protection of Human Research Subjects. Thus, this is an exempt study and there is no need for an IRB approval from our institution. The ethics committee waived the requirement of written informed consent for participation.

## Author Contributions

XL, JX, YS, and XZ contributed to the design, analysis, data interpretation, made revision for the manuscript, and approved the final edition.

## Conflict of Interest

The authors declare that the research was conducted in the absence of any commercial or financial relationships that could be construed as a potential conflict of interest.
