# Supplementary figures and images for: Prevalence and Associated Factors of Lupus in the United States: Third National Health and Nutritional Examination Survey (NHANES III)
Source: Front Med (Lausanne). 2020 May 27;7:213. doi: 10.3389/fmed.2020.00213 (PMC7266994; doi:10.3389/fmed.2020.00213)

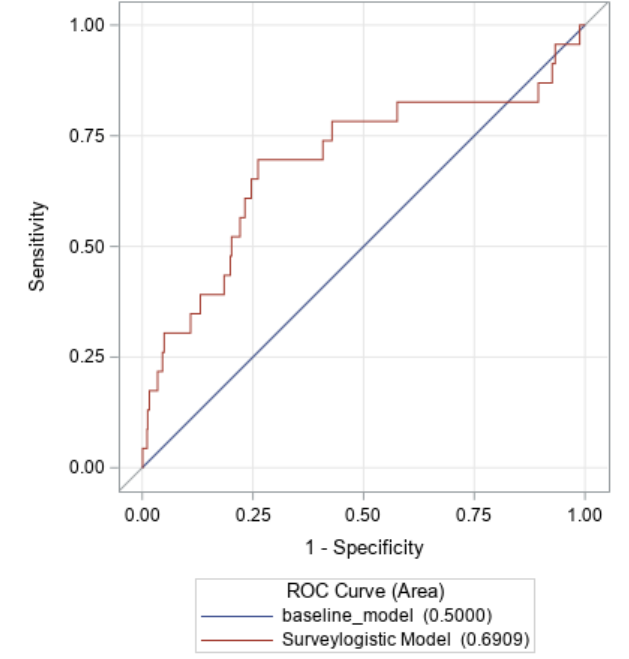


Supplemental Figure 1. ROC Curve for Multivariate Logistic Regression Model

Supplement: Supplementary file 1 [file Data_Sheet_1.docx]
